# A meta-analysis of XRCC1 single nucleotide polymorphism and susceptibility to gynecological malignancies

**DOI:** 10.1097/MD.0000000000028030

**Published:** 2021-12-17

**Authors:** Xue Qin Zhang, Li Li

**Affiliations:** Department of Gynecology and Oncology, Guangxi Medical University Cancer Hospital and Key Laboratory of Early Prevention and Treatment for Regional High Frequency Tumor, Ministry of Education, Nanning, China.

**Keywords:** gynecological malignancies, meta-analysis, polymorphism, susceptibility, X-ray repair cross complementation 1

## Abstract

**Background:**

Gynecological malignant tumor is a serious threat to women's health, cervical cancer, endometrial cancer and ovarian cancer are the most common. The eponymous protein encoded by the XRCC1 (X-ray repair cross complementation 1) gene is an important functional protein in the process of single-stranded DNA damage. Non-synonymous mutations of XRCC1 gene cause amino acid sequence changes that affect protein function and DNA repair ability, and may affect the interaction with other DNA repair proteins, leading to increased risk of tumor development. Many studies have assessed the association between XRCC1 gene polymorphism and the risk of cancer in the female reproductive system, but the results have been inconclusive. In this study, the relationship between XRCC1 Arg399Gln, Arg194Trp, Arg280His single nucleotide polymorphisms and susceptibility to gynecological malignancies was further explored by meta-analysis.

**Methods:**

English database: Pubmed, Medline, Excerpta Medica Database, Cochrance, etc; Chinese database: China national knowledge infrastructure, Wanfang Database, etc. STATA14 was used for statistical analysis, such as odd ratio (OR) value, subgroup analysis, heterogeneity test, sensitivity analysis, and publication bias.

**Results:**

In gynecologic cancers, the allele frequency difference of Arg399Gln case control group was statistically significant (GvsA: *P* = .007). There was no significant difference in allele frequency in the Arg194Trp and Arg280His case control groups (*P* = .065, 0.198). In different gene models, Arg399Gln was significantly correlated with gynecologic cancers susceptibility (GGvs AA: OR 0.91; 95% confidence interval [CI], 0.85 0.98); Arg194Trp was significantly correlated with gynecologic cancers susceptibility (CCvs TT: OR 0.94; 95% CI 0.88,1.00; CCvs CT: OR 0.97; 95% CI 0.90, 1.05); Arg280His was significantly correlated with gynecologic cancers susceptibility (GGvs AA: OR 0.98; 95% CI 0.94, 1.02; GGvs GA: OR 1.00;95% CI 0.97, 1.04). In the subgroup analysis, Arg399Gln and Arg194Trp were significantly correlated with gynecologic cancers susceptibility in the Asian race (*P* = .000, 0.049). In the analysis of different cancer subgroups, Arg399Gln and cervical cancer susceptibility were statistically significant (*P* = .039). Arg194Trp and endometrial cancer susceptibility were statistically significant (*P* = .033, 0.001).

**Conclusions:**

XRCC1 Arg399Gln, Arg194Trp, Arg280His single nucleotide polymorphisms were associated with gynecologic cancer susceptibility. Arg399Gln genotype was statistically significant in relation to cervical cancer susceptibility. Arg194Trp genotype was statistically significant in relation to endometrial cancer susceptibility.

## 1 Introduction

Gynecological malignant tumors are major diseases that seriously threaten women's health. Cervical cancer, endometrial cancer and ovarian cancer are the most common, and surgical treatment, radiotherapy and chemical therapy are the main treatment methods. Cervical cancer is the most common gynecologic tumor, with about 530,000 new cases and 260,000 deaths each year globally. With the popularization of cervical cancer screening, the incidence of cervical squamous epithelial lesions and cervical squamous cell carcinoma has decreased. However, the incidence of cervical adenocarcinoma is on the rise, with the proportion of cervical cancer rising from 5% to about 20%. The prognosis of early cervical cancer is relatively good, but there are still some problems in the diagnosis and treatment of cervical adenocarcinoma.^[1,2]^ At present, in western developed countries, the incidence of endometrial cancer ranks the first among malignant tumors of the female reproductive system, and its mortality is second only to ovarian cancer.^[3]^ Although endometrial cancer has a good prognosis in general, its increasing morbidity and mortality make its prevention and control situation increasingly severe.^[4]^ Ovarian cancer occupies the third place in the incidence of female genital malignancies, but its mortality is the highest. Because the early clinical symptoms of ovarian cancer are not obvious, the onset is insidious, and there is no reliable detection method, about 60% of ovarian cancer patients have advanced tumor and extensive metastasis at the first diagnosis, and the 5-year survival rate is only 40% to 45% according to statistics.^[5]^

Cancer is a multifactorial disease, and genetic factors are important factors affecting its genetic susceptibility. Single nucleotide polymorphisms (SNPs) are the most common genetic variation, accounting for about 90% of human genetic variation, and some loci have been shown to be related to gene phenotypes and tumor susceptibility.^[6,7]^ So it is important to find new molecular markers that are sensitive to cancer. X-ray cross complementary repair gene 1 (XRCC1) the size of about 33 KB, located in the chromosome 19 q13. 2-19 q13. 3, contains 17 exon, DNA damage repair mechanism is in the way of base excision repair of the important genes, and its main with a variety of enzymes including poly ADP ribose polymerase, DNA polymerase beta, and DNA ligase III form compounds involved in DNA repair process.^[8]^ Current XRCC1 polymorphism studies mainly focus on 3 nonsynonymous mutant SNPs, namely Arg194Trp (rs1799782), Arg280His (rs25489), Arg399Gln (rs25487). The relationship between XRCC1 polymorphism and susceptibility to malignant tumors (such as nasopharyngeal cancer, breast cancer, lung cancer, stomach cancer, liver cancer, pancreatic cancer, colorectal cancer, prostate cancer, glioma, etc) has been reported many times.^[9–15]^ Among the 3 non-synonymous mutated SNPs, Arg399Gln, and Arg194Trp were most correlated with cervical cancer susceptibility, but the conclusions were inconsistent. Therefore, this study used meta-analysis method to explore the relationship between XRCC1 Arg399Gln, Arg194Trp, Arg280His and susceptibility to 3 common female reproductive system tumors.

Many studies have assessed the association between polymorphism in the XRCC1 gene and the risk of cancer in the female reproductive system, but the results have been inconclusive. In this study, the relationship between XRCC1 Arg399Gln, Arg194Trp, Arg280His single nucleotide polymorphisms and susceptibility to gynecological malignancies was further explored by meta-analysis.

## 2 Methods and analysis

### 2.1 Protocol registration

The protocol was based on the Preferred Reporting Items for Systematic Reviews and Meta-Analyses Protocols (PRISMA-P) statement guidelines.

### 2.2 Inclusion and exclusion criteria

#### 2.2.1 Inclusion criteria

1. The literature was a study on the relationship between XRCC1 single nucleotide polymorphism and susceptibility to female reproductive tract malignancies.
2. Design experiments for case-control (patient-healthy population).
3. The research data were complete, and the frequency, odd ratio (OR) value and 95% CI of each genotype could be checked.
4. NOS (Newcastle-Ottawa Scale) score ≥7.

#### 2.2.2 Exclusion criteria

1. Literatures that do not meet the above inclusion criteria.
2. Summary, dissertation, conference summary, correspondence and letters.

### 2.3 Retrieval strategy

#### 2.3.1 Research objects

Published studies on the association between XRCC1 nucleotide polymorphism and susceptibility to cervical, endometrial, and ovarian cancer were collected, and the distribution differences of Arg399Gln, Arg194Trp, Arg280His in patients (case group) and healthy people (control group) were systematically evaluated.

#### 2.3.2 Retrieval database and method

English database: Pubmed, Medline, Excerpta Medica Database, Cochrance, Ovid; Chinese database: China national knowledge infrastructure, Wanfang database, Vip Chinese science and Technology journal. The retrieval time was set up until June 2020.

In The English database, the combination of subject words and free words is connected by “OR,” and between keywords is connected by “And.” Single nucleotide Polymorphism subject word “Polymorphism,” free words “Genetic Polymorphism, Polymorphism (Genetics), Genetic isms.” X ray cross complementing repair gene 1 subject word “XRCC1,” free word “X-ray cross-complementing 1 or Arg399Gln, Arg194Trp, Arg280His.” Female reproductive tract malignant tumor subject word “Female Reproductive system Cancer”; free word “Gynecological cancer.” Ovarian Cancer subject word “Ovarian Neoplasms,” free words “Ovarian Cancer, epithelial,” etc. Cervical Cancer subject word “Cervical Carcinoma,” free word “Cervical Cancer,” etc. Endometrial Cancer subject word “Endometrial Cancer,” free word “Endometrial Carcinoma,” etc. The Chinese database use “XRCC1,” “gene polymorphism,” “gynecological tumor,” “ovarian cancer,” “cervical cancer,” “endometrial cancer” and “susceptibility” as keywords, and there is no limitation in languages.

### 2.4 Literature screening, data extraction and quality evaluation

#### 2.4.1 Newcastle-ottawa scale

A self-made data collection table was adopted, literature selection and data entry were completed independently by two people, and disputes were settled through discussion and negotiation. The newcastle-Ottawa Quality Assessment Scale was used to evaluate the Quality of the included literature. The content of the Quality Assessment of the case-control study included 3 items, namely selection, exposure and comparability, and a total of 8 indicators.

Including:

1. Whether the definition of cases is adequate;
2. Representativeness of cases;
3. Comparison selection;
4. The definition of contrast;
5. Comparability between case and control;
6. Determination of exposure;
7. Whether the same determination method was used for case and control exposures;
8. No response rate.

The full score is 9, and those with scores ≥6 are of high quality, while those with scores less than 6 are of low quality. Quality evaluation (newcastle-Ottawa Quality Assessment Scale≥6) is the inclusion criterion.^[16]^

#### 2.4.2 Hardy-weinberg equilibrium

Hardy-weinberg equilibrium: when the gene is passed from generation to generation without the influence of evolution, the gene frequency and genotype frequency of the population will remain unchanged. *P* > .05 is the inclusion criteria.

#### 2.4.3 Linkage disequilibrium

Linkage Disequilibrium (LD): also called allelic association. In the linkage disequilibrium, there is a deviation between the probability of haplotype appearing on the same chromosome and the probability of random combination, which is the degree of LD and is caused by mutation. Theoretically, the size of LD is related to the distance between two sites. The smaller the distance is, the less the chance of recombination will be and the stronger the linkage imbalance will be.

In the case of Hardy-weinberg equilibrium, the probability of AB is: P (AB) = P (A)x P (B), If there is a linkage imbalance, then the probability of AB is P (AB). The difference between these two probabilities reflects the degree of chain imbalance, namely the index D. D = P (AB)- P (A)x P (B).

D = 0 means complete linkage equilibrium, when alleles on different loci are combined according to the random principle, the frequency of allele combination is equal to the product of the respective frequencies of alleles. At this time AB:Ab:aB:ab = 0.25:0.25:0.25:0.25, P (AB) = P (A)x P (B). D = 1 means complete linkage disequilibrium, AB: ab = 0.5:0.5, P (AB) = P (A)x P (B) + D. Standardized imbalance coefficient D^/^ = D/Dmax, when D > O, Dmax = minutes{P (A)P (B), P (a)P (b)}, when D < O, Dmax = minutes{P (A)P(b), P(a)P (B)}. *r*^2^ = D^2^/ P (A) P (a), P (B) P (b)}. *R*^2^ = 0 means that the chain is perfectly balanced, a random combination. *R*^2^ = 1 means that the linkage is completely unbalanced and there is no recombination, indicating that alleles at 2 loci have the same frequency, and the occurrence of an allele at 1 locus completely predicts the occurrence of corresponding alleles at the other locus.

When D is not 0, it indicates that there is linkage imbalance between the 2 genes. D is between 0 and 1, and the greater the D is, the higher the degree of linkage is. D > O means that the probability of the existence of two alleles (AB) on the same chromosome is greater than the probability of the occurrence of both alleles due to random distribution in the population. It is said that these 2 points are in the state of LD and there is an allelic association, which is of great significance for the study of gene correlation. For example, the linkage imbalance between SNP1 (G/A) and SNP2(C/T) was observed to be associated with disease susceptibility, and haplotype AC was A disease-related risk factor. D between 0-1 is the inclusion criteria.^[17–18]^

### 2.5 Statistical method

Five gene models were used: homozygous gene model (GG vs AA), heterozygous gene model (GG vs GA), dominant gene model (GG vs GA + AA), recessive gene model (AA vs GG + GA), and allelic gene model (G vs A).

STATA14 was used for data analysis. The specificity and sensitivity were measured by combined OR, the interval estimation was expressed by 95% confidence interval [CI], and *P* < .05 was considered statistically significant. The heterogeneity was analyzed by Q test. When *P* < .10, the heterogeneity was indicated, and the random effect model was used; otherwise, the fixed effect model was used. The heterogeneity was represented by *I*^2^. When *I*^2^ > 50% or *P* < .10, the random effect model was used; otherwise, the fixed effect model was used.^[19]^ Subgroup analysis was performed according to different conditions. If necessary, sensitivity analysis was performed and funnel plot was used to detect publication bias.

Because of the difference of the transcriptome sequencing expression is analysis of gene expression values of a large number of independent statistical hypothesis test, the problems will be false positives, so in the process of analyzing differentially expressed, the recognized Benjamini - Hochberg correction method of hypothesis testing have been the original significance *P* values for correction, and eventually the FDR (false discovery rate) as the key indicators of screening differentially expressed genes. FDR < 0.01 or 0.05 is generally taken as the default standard.

## 3 Results

### 3.1 Data collection and analysis

#### 3.1.1 Literature search results

**Figure 1:**
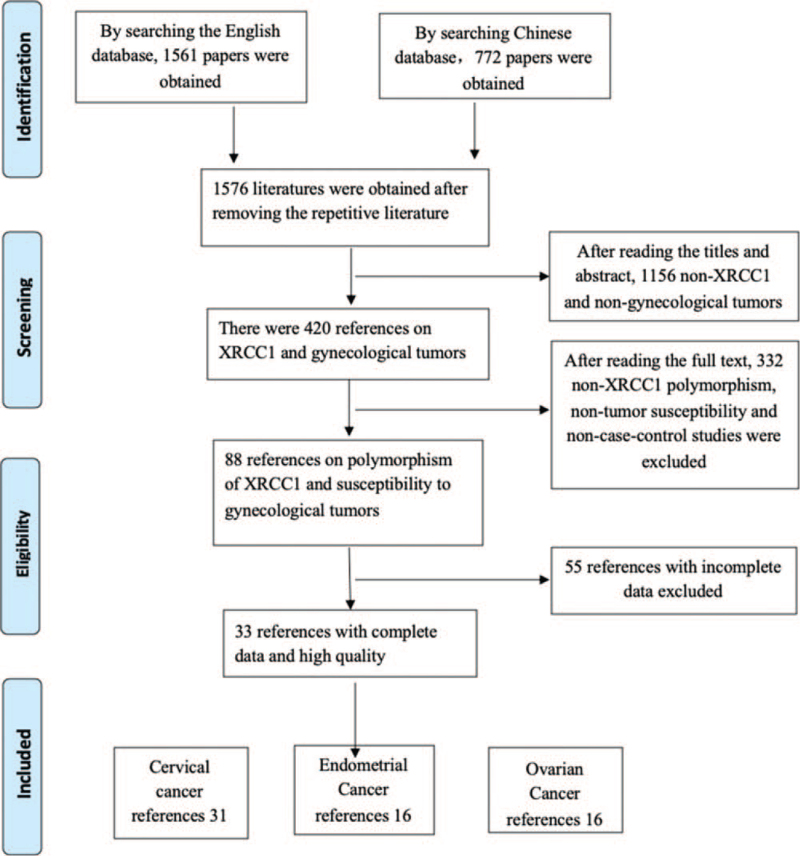
Literature retrieval and screening process.

Two thousand three hundred thirty three references were retrieved from various databases, and 1576 references were obtained after reading titles and removing duplicates. Reading abstracts excluded 1156 non-relevant literatures that did not meet the inclusion criteria, and 420 of them were obtained. After reading the full text, 55 incomplete literatures were excluded, and 33 literatures were finally included. The retrieval process is shown in Figure 1.

#### 3.1.2 The basic characteristics and quality evaluation of the included literature

Thirty three articles were included as case control studies, with a total of 6233 cases in the case group and 8555 cases in the control group. The samples were all from venous blood and were tested by different genetic methods. Khokhrin^[31]^ only gave the genotype distribution frequency, and Khrunin A V^[54]^ published its gene sequencing results and typing, but did not control the influence of other confounding factors. Ma Ning^[25]^ genotype data were incomplete, and the literature was excluded. **Baseline characteristics and quality scores of references**^[20–53]^**are shown in**Table 1.

**Table 1:** Characteristics and quality evaluation of included literature.

Number	First author	Type of cancer	Year	Country (region)	Ethnicity	Case/control	Source of controls	Platform	NOS	Genotyped SNPs
1	Joo	Cervical cancer	2016	Korea	Asian	478/922	Popupation based(PB)	Taqman	8	Arg194Trp Arg280His Arg399Gln
2	Bajpai	Cervical cancer	2015	India	Asian	65/68	Hospital based (HB)	PCR-RFLP	8	
3	Zhang	Cervical cancer	2012	China	Asian	80/177	Popupation based	SNPstream	8	
4	Huang	Cervical cancer	2007	China	Asian	539/800	Popupation based	MA-PCR	8	
5	Wu	Cervical cancer	2004	China (Taiwan)	Asian	100/196	Popupation based	PCR-RFLP	7	
6	Ma ning	Cervical cancer	2019	China	Asian	100/200	Hospital based	Taqman	6	Arg194Trp Arg399Gln
7	Monteiro	Ovarian cancer	2014	Brazil	Latino	70/70	Popupation based	PCR-RFLP	7	
8	Hosono	**Endometrial cancer**	2013	Japan	Asian	91/261	Hospital based	Taqman	8	
9	Djansugurova	Cervical cancer	2013	Kazakhstan	Asian	217/160	Popupation based	PCR-RFLP	8	
10	Fan	Cervical cancer	2013	China	Asian	235/350	Popupation based	MA-PCR	7	
11	Sobczuk	**Endometrial cancer**	2012	Poland	European	94/114	N/A	PCR-RFLP	8	
12	Khokhrin	Ovarian cancer	2012	Russian	European	104/298	Popupation based	PCR-RFLP	7	
13	Barbisan	Cervical cancer	2011	Argentine	Latino	103/114	N/A	PCR-RFLP	8	
14	Settheetham-I Wannapa	Cervical cancer	2011	Thailand	Asian	111/118	Popupation based	PCR-RFLP	8	
15	Farkasova	Cervical cancer	2008	Slovakia	European	17/30	Hospital based	PCR-RFLP	8	
16	Sonali Verma	Ovarian cancer	2019	India	European	130/150	Popupation based	PCR-RFLP	8	Arg399Gln
17	Abbas M	Cervical cancer	2019	India	European	260/265	Popupation based	PCR-RFLP	7	
18	Al-harbi	Cervical cancer	2017	Saudi Arabia	Asian	232/313	Hospital based	PCR-RFLP	7	
19	Chen	**Endometrial cancer**	2016	China	Asian	108/ 110	Hospital based	PCR-RFLP	7	
20	Zhou	Cervical cancer	2015	China	Asian	102/112	Hospital based	MA-PCR	7	
21	Malisic	Ovarian cancer	2015	Serbia	European	50/78	Popupation based	PCR-RFLP	8	
22	Alsbeih	Cervical cancer	2013	Saudi Arabia	Asian	100/100	N/A	Sequencing	8	
23	Cincin	**Endometrial cancer**	2012	Turkey	European	104/158	Hospital based	PCR-RFLP	7	
24	Samulak	**Endometrial cancer**	2011	European	Asian	456/300	Hospital based	PCR-RFLP	7	
25	Roszak	Cervical cancer	2011	European	Asian	189/308	Popupation based	PCR-RFLP	8	
26	Makowska	Endometrial cancer	2011	European	Asian	150/150	N/A	PCR-RFLP	8	
27	Ma	Cervical cancer	2011	China	Asian	200/200	Popupation based	PCR-RFLP	8	
28	Xiao	Cervical cancer	2010	China	Asian	162/183	Popupation based	PCR-RFLP	7	
29	Jiang	Cervical cancer	2009	China	Asian	436/503	Popupation based	PCR-RFLP	7	
30	Wang S	Cervical cancer	2009	Costa Rica	Latino	469/452	Popupation based	Taqman	8	
31	Jakubowska	Ovarian cancer	2009	Poland	European	143/280	Popupation based	PCR-RFLP	8	
32	Niwa	Cervical cancer	2005	Japan	Asian	131/320	Popupation based	PCR-RFLP	8	
33	Michalska	Ovarian cancer	2015	European	Asian	720/720	Hospital based	PCR-RFLP	8	Arg194Trp
34	Wang X	Cervical cancer	2010	China	Asian	123/175	Hospital based	PCR-RFLP	7	Arg194Trp Arg280His

#### 3.1.3 Genotype distribution of the included literature

In this study, 3 SNP loci of XRCC1 gene Arg399Gln, Arg194Trp and Arg280His were genotyped, and the linkage imbalance relationship between loci was analyzed. The genotype distribution frequency of the case group and the control group, among which, whether the genotype distribution conforms to the Hardy-weinberg equilibrium (*P* > .05) is shown in Table 2. Results: Most of the genotypes of the 3 SNP sites were distributed in Hardy-weinberg equilibrium.

**Table 2:** Genotype distribution of included XRCC1 Arg399Gln, Arg194Trp, Arg280His in gynecological cancer.

Author	Cancer type	Case/control	Genotype distribution						HW-E	
			case			control			*P* > .05	
Arg399Gln (G > A)			(Arg/Arg) GG	(Arg/Gln) GA	(Gln/Gln ) AA	(Arg/Arg ) GG	(Arg/Gln ) GA	(Gln/Gln ) AA	Case	Control
Ma ning 2019	Cervical cancer	100/200	46	54	118	82				
Abbas M 2019	Cervical cancer	260/265	109	112	39	141	102	22	0.251	0.561
Al-harbi 2017	Cervical cancer	232/313	114	89	29	177	121	15	0.083	0.321
Joo 2016	Cervical cancer	478/922	257	194	27	500	354	68	0.219	0.625
Bajpai 2015	Cervical cancer	65/68	12	22	31	23	33	12	0.036	0.978
Zhou 2015	Cervical cancer	102/112	40	41	21	61	33	18	0.091	0.001
Alsbeih 2013	Cervical cancer	100/100	52	34	14	59	40	1	0.040	0.398
Djansugurova 2013	Cervical cancer	217/160	78	119	20	66	90	4	0.008	0.411
Fan 2013	Cervical cancer	235/350	33	137	65	11	224	115	0.004	0.000
Zhang 2012	Cervical cancer	80/177	43	31	6	109	58	10	0.900	0.538
Barbisan 2011	Cervical cancer	103/114	54	31	18	37	59	18	0.001	0.490
Ma 2011	Cervical cancer	200/200	108	76	16	133	55	12	0.610	0.610
Roszak 2011	Cervical cancer	189/308	49	101	39	116	152	40	0.324	0.371
Wannapa 2011	Cervical cancer	111/118	66	41	4	69	44	5	0.437	0.539
Xiao 2010	Cervical cancer	162/183	91	56	15	94	68	21	0.148	0.116
Jiang 2009	Cervical cancer	436/503	228	184	24	268	194	41	0.092	0.482
Wang S 2009	Cervical cancer	457/442	225	198	34	195	195	52	0.286	0.761
Farkasova 2008	Cervical cancer	18/30	8	9	1	11	17	2	0.450	0.179
Huang 2007	Cervical cancer	539/800	289	203	47	528	235	37	0.189	0.104
Niwa 2005	Cervical cancer	131/320	69	49	13	185	109	26	0.333	0.088
Wu 2004	Cervical cancer	100/196	54	38	8	114	73	9	0.719	0.531
Chen 2016	**Endometrial cancer**	108/ 110	46	46	16	65	36	9	0.424	0.222
Hosono 2013	Endometrial cancer	91/261	57	33	1	137	106	18	0.110	0.681
Cincin 2012	Endometrial cancer	104/158	86	13	5	138	20	0	0.000	0.204
Sobczuk 2012	Endometrial cancer	94/114	27	45	22	43	48	23	0.699	0.161
Samulak 2011	Endometrial cancer	456/300	72	90	294	72	144	84	0.000	0.505
Makowska 2011	Endometrial cancer	150/150	41	73	36	64	68	18	0.754	0.992
**Sonali Verma** 2019	Ovarian cancer	130/150	80	1	49	105	1	44	0.000	0.000
**Malisic** 2015	Ovarian cancer	50/78	29	16	5	30	21	27	0.234	0.000
**Monteiro** 2014	Ovarian cancer	70/70	35	28	7	35	30	5	0.690	0.676
**Khokhrin** 2012	Ovarian cancer	104/298	48	45	11	134	131	33	0.925	0.908
**Jakubowska** 2009	Ovarian cancer	143/280	52	68	23	100	138	42	0.922	0.617

Arg194Trp (C > T)			(Arg/Arg) CC	(Arg/Trp) CT	(Trp/Trp) TT	(Arg/Arg) CC	(Arg/Trp) CT	(Trp/Trp) TT	Case	Control
Ma ning 2019	Cervical cancer	100/200	47	53		93	107			
Joo 2016	Cervical cancer	478/922	219	211	48	414	406	102	0.786	0.869
Bajpai 2015	Cervical cancer	65/68	11	16	38	44	11	13	0.001	0.000
Djansugurova 2013	Cervical cancer	217/160	163	48	6	105	40	15	0.291	0.001
Fan 2013	Cervical cancer	235/350	83	128	24	149	196	5	0.013	0.000
Zhang 2012	Cervical cancer	80/177	41	31	8	87	71	19	0.554	0.434
Barbisan 2011	Cervical cancer	103/114	79	20	4	98	12	4	0.785	0.000
Wannapa 2011	Cervical cancer	111/118	53	49	9	65	51	2	0.617	0.023
Wang X 2010	Cervical cancer	123/175	65	47	11	114	54	7	0.554	0.849
Farkasova 2008	Cervical cancer	17/30	14	3	0	24	6	0	0.690	0.542
Huang 2007	Cervical cancer	539/800	241	220	78	407	330	63	0.018	0.731
Wu 2004	Cervical cancer	100/196	48	43	9	87	93	16	0.886	0.196
Hosono 2013	Endometrial cancer	91/261	30	46	15	125	113	23	0.708	0.722
Sobczuk 2012	Endometrial cancer	94/114	89	5	0	103	11	0	0.791	0.588
Michalska MM 2015	Ovarian cancer	720/720	180	360	180	190	334	196	1	0.053
**Monteiro** 2014	Ovarian cancer	70/70	61	9	0	57	12	1	0.565	0.690
**Khokhrin** 2012	Ovarian cancer	104/298	94	0	10	254	43	1	0.000	0.562

Arg280His (G > A)			(Arg/Arg) GG	(Arg/His) GA	(His/His) AA	(Arg/Arg) GG	(Arg/His) GA	(His/His) AA		
Joo 2016	Cervical cancer	478/922	371	100	7	717	195	10	0.930	0.418
Bajpai 2015	Cervical cancer	65/68	20	6	39	48	7	13	0.000	0.000
Zhang 2012	Cervical cancer	80/177	68	11	1	142	34	1	0.480	0.494
Wang X 2010	Cervical cancer	123/175	96	19	8	145	26	4	0.000	0.043
Huang 2007	Cervical cancer	539/800	416	117	6	620	171	9	0.482	0.463
Wu 2004	Cervical cancer	100/196	74	24	2	140	55	1	0.973	0.071

HWE = hardy-weinberg equilibrium, XRCC1 = X-ray repair cross complementation 1.

Linkage disequilibrium analysis was carried out between 3 SNP sites in pairs in 5 literatures and 2 SNP sites in 10 literatures. Three SNPS can form 6 haplotypes:GCG, GCA, GTA, ACG, ACA, ATG, and ATA. According to the analysis of haploview software, the linkage disequilibrium coefficient D > 0 was found, indicating the strong linkage disequilibrium existed in the 3 SNP sites of XRCC1 gene. Conclusion: The 3 SNP sites of Arg399Gln, Arg194Trp and Arg280His of XRCC1 gene showed complete linkage disequilibrium.

### 3.2 Statistical analysis

#### 3.2.1 Meta-analysis data

Five gene models were used for analysis. The heterogeneity was represented by *I*^2^, and when *I*^2^ > 50% or *P* < .10, there was heterogeneity, and a random effect model was used. The relationship between different genotypes of the 3 loci and cancer susceptibility was heterogeneous, and the random-effect model was used. As shown in Tables 3–5. *P* < .05 indicated that the genotype distribution frequency difference in the case control group was statistically significant, and the polymorphism of this site was correlated with cancer susceptibility.

**Table 3:** Summary ORs of the *XRCC1* Arg399Gln polymorphism and gynecologic cancer risk.

		Homozygous genetic model			Heterozygous genetic model			Dominant gene model			Recessive gene model			Allelic model		
Variables	Studies	OR (95% CI)	*P* value	*I*^2^, %	OR (95% CI)	*P* value	*I*^2^, %	OR (95% CI)	*P* value	*I*^2^, %	OR (95% CI)	*P* value	*I*^2^, %	OR (95% CI)	*P* value	^*I*2^, %
Arg399Gln		GG vs AA			GG vs GA			*GG* vs GA* +* AA			GG* +* GA vs AA			G vs A		
total	31	0.91 (0.85, 0.98)	.009	90.6	0.97 (0.92,1.02)	.231	62.1	0.92 (0.89, 0.96)	.000	70.2	1.33 (1.17, 1.52)	.000	49.0	0.93 (0.88, 0.98)	.007	89.8
Ethnicity																
Asian	20	0.93 (0.91, 0.95)	.000	87.6	0.94 (0.93–0.98)	.002	63.0	0.89 (0.86, 0.93)	.000	71.7	1.57 (1.31, 1.89)	.000	43.9	0.89 (0.83, 0.95)	.001	91.7
Non-Asian	11	0.99 (0.95, 1.04)	.744	69.5	1.02 (0.96, 1.08)	.539	47.4	1.01 (0.94, 1.08)	.864	64.0	1.07 (0.88, 1.31)	.486	40.9	1.00 (0.94, 1.07)	.901	75.4
Agreement with HWE	27	0.93 (0.91, 0.95)	.000	82.8	0.95 (0.92, 0.99)	.005	48.5	0.91 (0.88, 0.95)	.000	62.4	1.48 (1.34, 1.63)	.000	71.8	0.91 (0.86, 0.97)	.001	90.1
Cancer type																
Cervical cancer	20	0.92 (0.85, 1.00)	.039	90.9	0.95 (0.91–0.99)	.284	65.6	0.93 (0.89, 0.97)	.000	73.3	1.54 (1.27, 1.86)	0.000	35.2	0.93 (0.88, 0.99)	.027	89.4
Endometrial cancer	6	0.79 (0.60, 1.03)	.083	96.9	0.98 (0.90–1.07)	.622	67.6	0.83 (0.76, 0.92)	.000	79.8	1.47 (1.08, 2.01)	.016	59.6	0.84 (0.68, 1.03)	.089	95.5
Ovarian cancer	5	1.03 (0.89, 1.20)	.674	70.8	1.02 (0.93–1.13)	.951	0.00	1.01 (0.90,1.13)	.852	44.0	1.00 (0.79, 1.26)	.996	60.8	1.03 (0.91, 1.17)	.649	77.4
Sample size																
≥500	**7**	0.96 (0.93–0.99)	.005	92.4	0.97 (0.92, 1.02)	.186	81.6	0.93 (0.88, 0.98)	.004	85.3	1.35 (1.20, 1.52)	.000	91.0	0.88 (0.77,1.00)	.053	96.5
<500	24	0.93 (0.90–0.96)	.000	74.5	0.95 (0.91, 1.00)	.040	50.8	0.92 (0.87, 0.96)	.001	58.8	1.33 (1.17, 1.52)	.000	49.0	0.95 (0.91, 0.99)	.026	71.7

CI = confidence interval, OR = odd ratio, XRCC1 = X-ray repair cross complementation 1.

**Table 4:** Summary ORs of the *XRCC1* Arg194Trp polymorphism and gynecologic cancer risk.

		Homozygous genetic model			Heterozygous genetic model			Dominant gene model			Recessive gene model			Allelic model		
Variables	Studies	OR (95% CI)	*P* value	*I*^2^, %	OR (95% CI)	*P* value	*I*^2^, %	OR (95% CI)	*P* value	*I*^2^, %	OR (95% CI)	*P* value	*I*^2^, %	OR (95% CI)	*P* value	*I*^2^, %
Arg194Trp		CC vs TT			CC vs CT			CC vs CT + TT			CC + CT vs TT			C vs T		
total	16	0.94 (0.88, 1.00)	.037	83.7	0.97 (0.90, 1.05)	.000	80.4	0.94 (0.87, 1.02)	.165	75.5	1.58 (1.08, 2.31)	.018	81.8	0.96 (0.91, 1.00)	.065	83.5
Ethnicity																
Asian	12	0.92 (0.84,1.00)	.049	84.2	0.96 (0.90, 1.03)	.259	50.1	0.91 (0.82, 1.02)	.092	78.2	1.51 (1.03, 2.20)	.035	83.6	0.94 (0.88, 1.00)	.050	87.2
Non-Asian	**4**	0.97 (0.90, 1.05)	.479	81.9	1.04 (0.87, 1.23)	.691	87.6	1.01 (0.92, 1.11)	.807	47.2	2.48 (0.20, 3.40)	.482	77.3	0.99 (0.95, 1.03)	.531	22.3
agreement with HWE	11	0.95 (0.90, 1.01)	.091	68.9	1.00 (0.91, 1.10)	.967	82.3	0.98 (0.91, 1.06)	.620	60.8	1.35 (0.95, 1.92)	.096	74.8	0.97 (0.94, 1.01)	.153	69.7
Cancer type																
Cervical cancer	**11**	0.93 (0.85, 1.01)	0.037	86.0	0.96 (0.90, 1.02)	.128	33.9	0.92 (0.82, 1.02)	0.119	60.8	1.56 (0.98, 2.50)	.061	80.1	0.94 (0.87, 1.00)	.684	85.7
Endometrial cancer	2	0.79 (0.63, 0.98)	.033	0.0	0.90 (0.54, 1.51)	.001	90.9	0.86 (0.43, 1.71)	.664	94.3	1.87 (1.02, 3.43)	.043	81.2	0.93 (0.65, 1.33)	.684	96.1
Ovarian cancer	3	0.97 (0.89, 1.06)	.560	76.5	1.05 (0.81,1.35)	.000	93.2	1.04 (0.95, 1.13)	0.379	32.3%	2.21 (0.19, 25.79)	.527	82.7	1.00 (0.97, 1.04)	0.065	2.7
Sample size																
≥500	**4**	0.92 (0.82, 1.04)	.168	84.4	0.96 (0.90, 1.03)	.222	0.00	0.93 (0.85, 1.01)	.098	30.8	1.52 (0.89, 2.60)	.122	90.4	0.96 (0.89, 1.02)	.181	76.7
<500	12	0.94 (0.88, 1.00)	.131	86.0	0.98 (0.88, 1.08)	.642	83.3	0.95 (0.85, 1.05)	.306	79.6	1.62 (0.90, 2.91)	.108	72.6	0.95 (0.89, 1.01)	.130	86.5

CI = confidence interval, OR = odd ratio, XRCC1 = X-ray repair cross complementation 1.

**Table 5:** Summary ORs of the *XRCC1* Arg280His polymorphism and gynecologic cancer risk.

Variables	Studies	Homozygous genetic model			Heterozygous genetic model			Dominant gene model			Recessive gene model			Allelic model		
		OR (95% CI)	*P* value	*I*^2^, %	OR (95% CI)	*P* value	*I*^2^, %	OR (95% CI)	*P* value	*I*^2^, %	OR (95% CI)	*P* value	*I*^2^, %	OR (95% CI)	*P* value	*I*^2^, %
Arg280His		GG vs AA			GG vs GA			GG vs GA + AA			GG + GA vs AA			G vs A		
Total (Asian)	6	0.98 (0.94, 1.02)	.001	88.3	1.00 (0.97, 1.04)	.000	0.00	0.98 (0.89, 1.06)	.504	74.4	2.21 (1.44, 3.40)	.000	8.3	0.96 (0.91,1.02)	.198	87.7
agreement with HWE	4	1.00 (0.99, 1.01)	.457	0.00	1.01 (0.97,1.05)	.624	0.00	1.01 (0.97,1.05)	.759	0.00	1.33 (0.69, 2.55)	.398	0.0	1.00 (0.97,1.02)	.694	5.7
Sample size																
≥500	**2**	1.00 (0.99, 1.01)	.702	0.0	1.00 (0.96, 1.04)	.956	0.0	1.00 (0.96, 1.04)	.887	0.0	1.71 (0.58, 2.36)	.664	0.0	1.00 (0.98,1.02)	.817	0.0
<500	4	0.90 (0.79, 1.04)	.147	95.8	1.002 (0.95, 1.08)	.645	0.0	0.89 (0.73, 1.10)	.284	85.7	3.09 (1.94, 4.92)	.000	0.0	0.88 (0.75,1.02)	.096	93.7

CI = confidence interval, OR = odd ratio, XRCC1 = X-ray repair cross complementation 1.

The allele frequency difference of Arg399Gln case control group was statistically significant (G vs A: *P* = .007 < .05). There was no significant difference in allele frequency between Arg194Trp and Arg280His cases (*P* > .05). In different gene models, Arg399Gln was significantly correlated with gynecologic cancer susceptibility (GG vs AA: OR 0.91; 95% CI 0.85, 0.98). There was no statistically significant correlation between Arg399Gln and gynecologic cancer susceptibility (GG vs GA: OR 0.97; 95% CI 0.92, 1.02). Arg194Trp was statistically significant in relation to susceptibility to gynecologic cancer (CC vs TT: OR 0.94; 95% CI, 0.88 1.00; CC vs CT: OR 0.97; 95% CI 0.90, 1.05). Arg280His was correlated with gynecologic cancer susceptibility with statistical significance (GG vs AA: OR 0.98; 95% CI, 0.94 1.02; GG vs GA: OR 1.00; 95% CI 0.97, 1.04).

#### 3.2.2 Subgroup analysis data

In the subgroup analysis, Arg399Gln was significantly correlated with gynecologic cancer susceptibility in the Asian race (*P* < .05). Arg194Trp was significantly associated with gynecologic cancer susceptibility in Asian ethnicity (*P* < .05). All ethnic groups of Arg280His documents were Asian and no subgroup analysis was performed. In cervical, ovarian and endometrial cancers, Arg399Gln was statistically significant with cervical cancer susceptibility (*P* < .05 in each gene model). Arg194Trp was statistically significant with endometrial cancer susceptibility (CC vs TT, CC vs CT, *P* < .05).

All significance *P* values (*P* < .05) were sorted, benjamin-Hochberg corrected, FDR < 0.05, the probability of false positive was low, *P* value was statistically significant. The meta-analysis and subgroup analysis of forest map are shown in Figure 2  .

**Figure 2:**
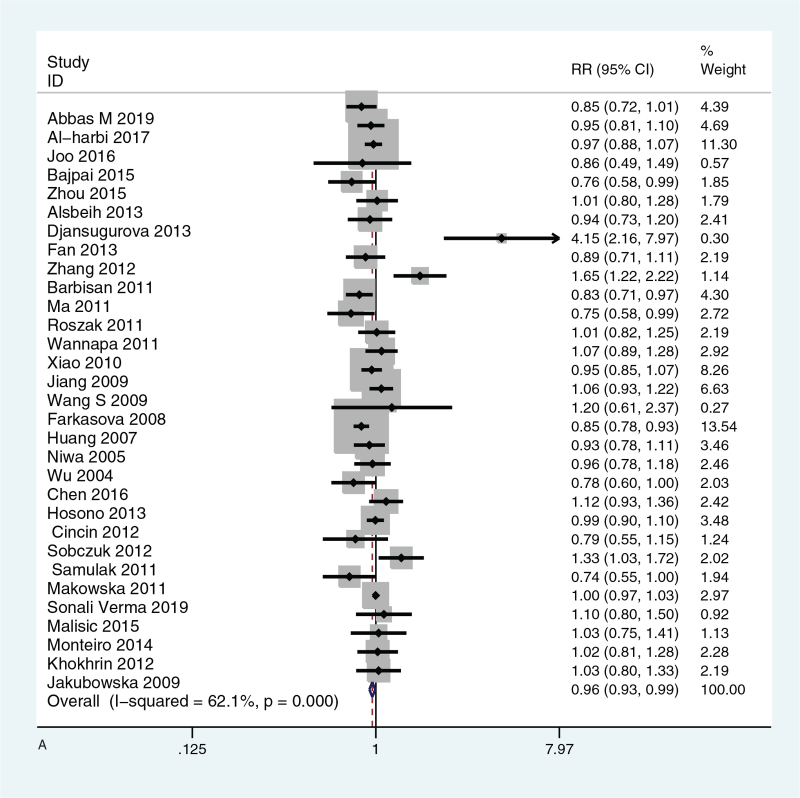
(A) Meta-analysis of forest map of the relationship between XRCC1 Arg399Gln gene model GGvs GA and susceptibility to gynecological cancer; (B) Forest map of subgroup analysis of the relationship between XRCC1 Arg399Gln GGvs GA and susceptibility to gynecological cancer; (C) Forest map of subgroup analysis of the relationship between XRCC1 Arg194Trp CCvs CT and susceptibility to gynecological cancer.

**Figure 2 (Continued):**
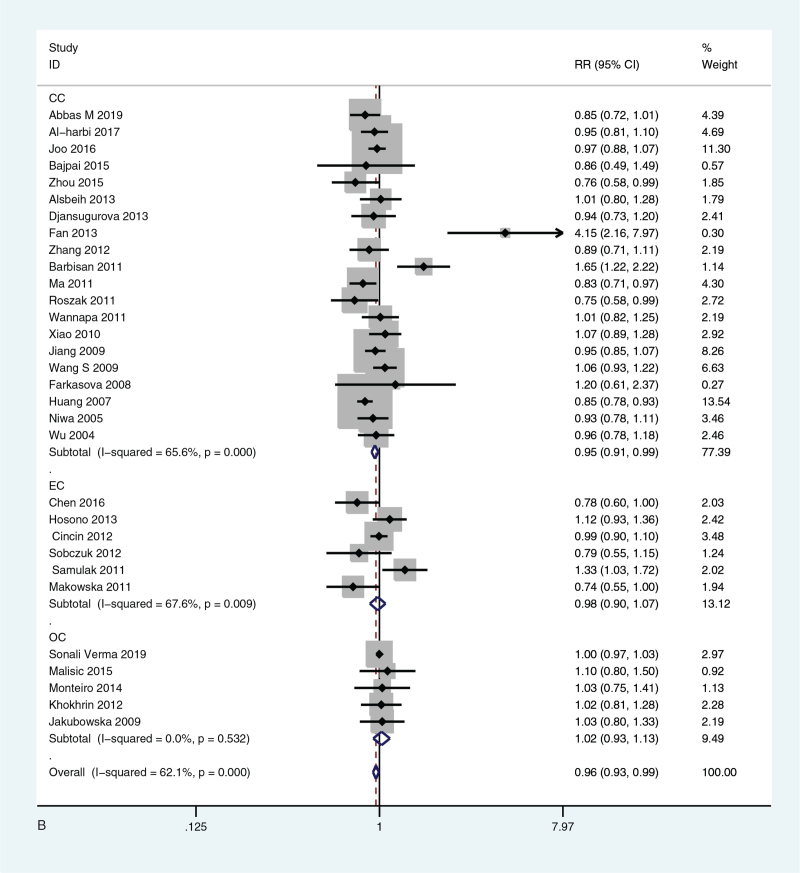
(A) Meta-analysis of forest map of the relationship between XRCC1 Arg399Gln gene model GGvs GA and susceptibility to gynecological cancer; (B) Forest map of subgroup analysis of the relationship between XRCC1 Arg399Gln GGvs GA and susceptibility to gynecological cancer; (C) Forest map of subgroup analysis of the relationship between XRCC1 Arg194Trp CCvs CT and susceptibility to gynecological cancer.

**Figure 2 (Continued):**
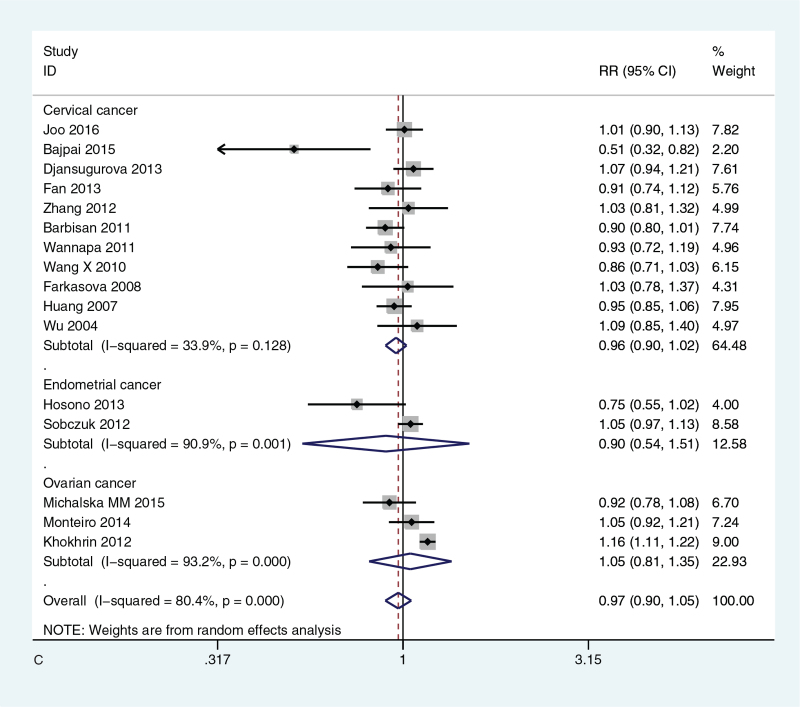
(A) Meta-analysis of forest map of the relationship between XRCC1 Arg399Gln gene model GGvs GA and susceptibility to gynecological cancer; (B) Forest map of subgroup analysis of the relationship between XRCC1 Arg399Gln GGvs GA and susceptibility to gynecological cancer; (C) Forest map of subgroup analysis of the relationship between XRCC1 Arg194Trp CCvs CT and susceptibility to gynecological cancer.

### 3.3 Sensitivity analysis

Sensitivity analysis was performed on the relationship between XRCC1 Arg399Gln GGvs AA and susceptibility to gynecologic cancer. After 1 article was removed in turn, no significant changes were found in the effect scale of 31 articles, and the results were still within 95% CI (95% confidence interval).

In the sensitivity analysis of Arg194Trp CCvs TT in XRCC1 and susceptibility to gynecologic cancer, after 1 article was removed in turn, no significant changes were found in the effect scale of 16 articles, and the results were still within 95% CI (95% confidence interval).

Sensitivity analysis of the relationship between XRCC1 Arg194Trp CCvs TT and susceptibility to gynecologic cancer. After 1 article was removed in turn, no significant changes in the effect scale were found in 6 articles, and the results were still within 95% CI. As shown in Figure 3 .

**Figure 3:**
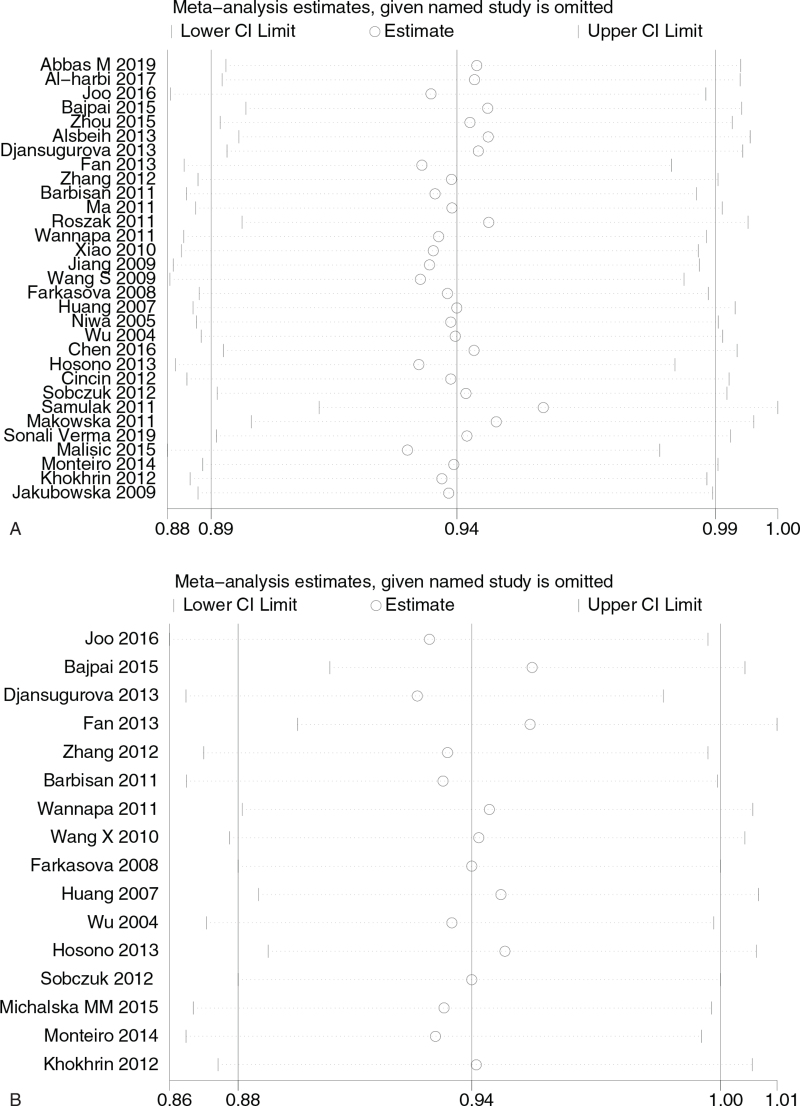
(A) Sensitivity analysis of the relationship between XRCC1 Arg399Gln GGvs AA and gynecological cancer susceptibility; (B) Sensitivity analysis of the relationship between XRCC1 Arg194Trp CCvs TT and gynecological cancer susceptibility; (C) Sensitivity analysis of the relationship between XRCC1 Arg280His GGvs GA and gynecological cancer susceptibility.

**Figure 3 (Continued):**
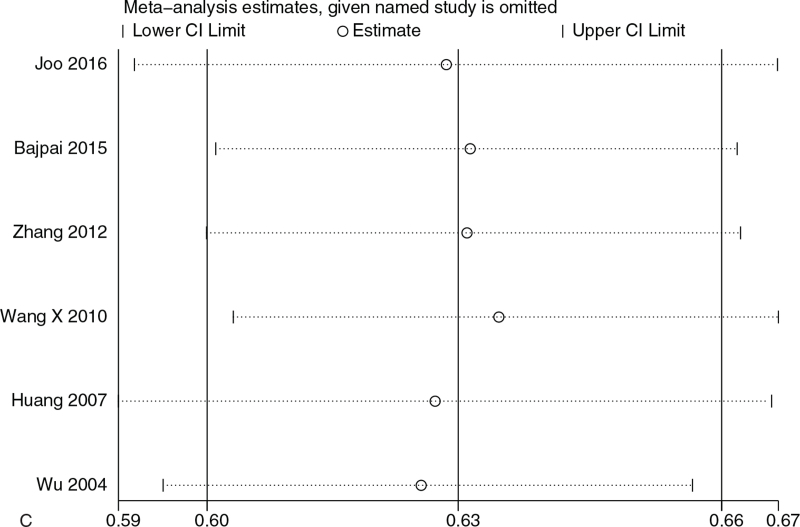
(A) Sensitivity analysis of the relationship between XRCC1 Arg399Gln GGvs AA and gynecological cancer susceptibility; (B) Sensitivity analysis of the relationship between XRCC1 Arg194Trp CCvs TT and gynecological cancer susceptibility; (C) Sensitivity analysis of the relationship between XRCC1 Arg280His GGvs GA and gynecological cancer susceptibility.

### 3.4 Publication bias

**Figure 4:**
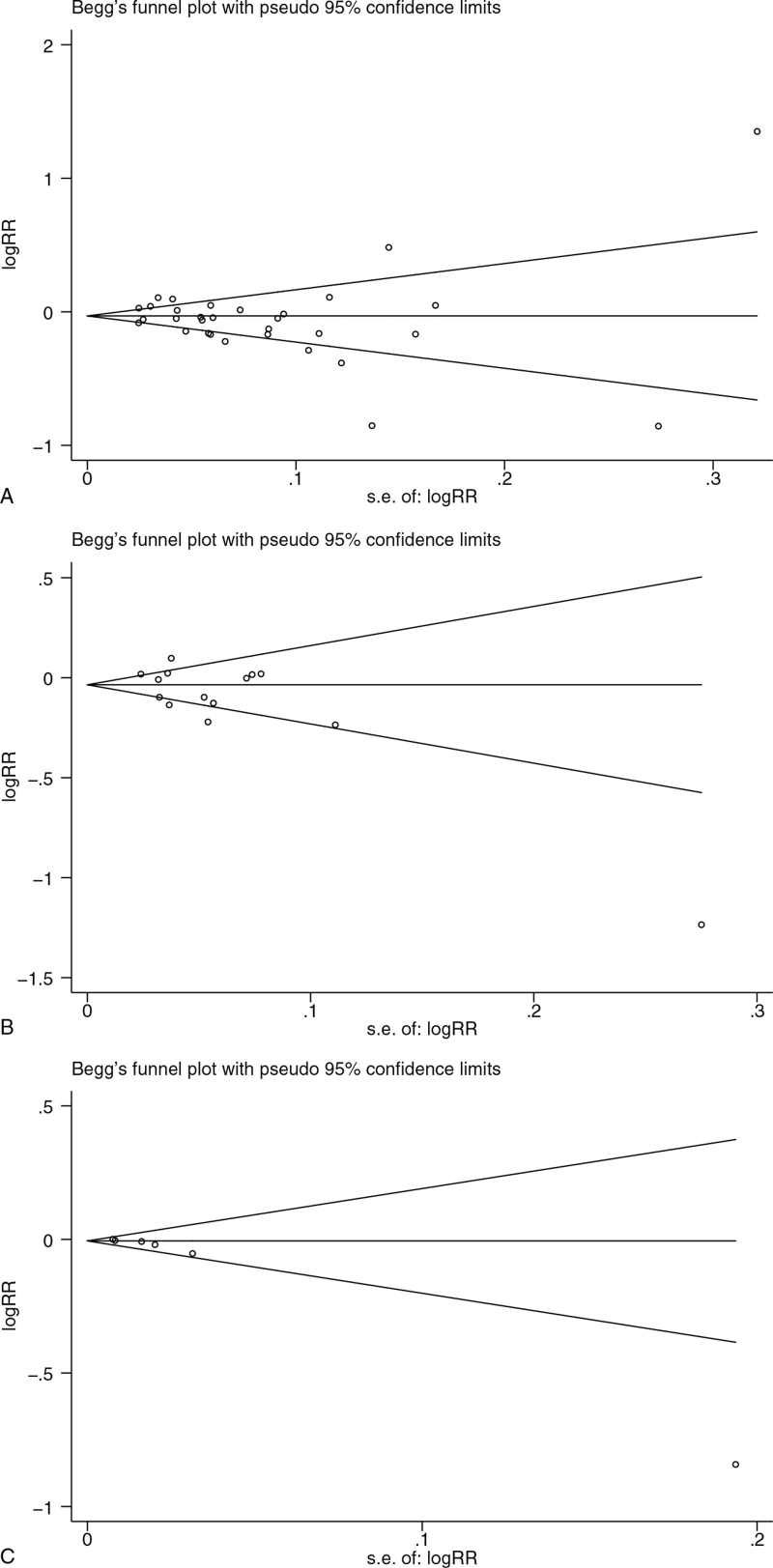
(A) Funnel plot of XRCC1 Arg399Gln GGvs AA and gynecological cancer susceptibility; (B) Funnel plot of XRCC1 Arg194Trp CCvs TT and gynecological cancer susceptibility; (C) Funnel plot of XRCC1 Arg280His GGvs GA and gynecological cancer susceptibility.

The funnel plots of XRCC1 Arg399Gln GGvs AA and the susceptibility of the gynecologic cancer showed certain publication bias, and the funnel plot nodes formed relatively uniform funnel shape, indicating small publication bias, as shown in Figure 4. Begg's Test N = 31, *z* = 0.99, Pr > |z| = 0.32, Egger test *P* > |t| = 0.187, *P* > .05 indicates no significant publication bias.

Funnel plot of Arg194Trp CCvs TT in XRCC1 and the susceptibility of the gynecologic cancer showed significant publication bias, as shown in Figure 4. Begg Test N = 14, *z* = 1.31, Pr > |z| = 0.189. Egger test *P* > |t| = 0.056, *P* > .05 indicates no significant publication bias.

The funnel plot of Arg280His GGvs GA of XRCC1 and the susceptibility of the gynecologic cancer showed significant publication bias, as shown in Figure 4. Begg Test N = 6, z = 2.63, Pr > |z| = 0.009. Egger test P > |t| = 0.017, P < 0.05 indicates publication bias. All data are shown in Table 6.

**Table 6:** Egger test (XRCC1 and susceptibility to gynecological cancer).

XRCC1	Std_Eff	Coef.	Std. Err.	t	*P* > |t|	[95% CI]
Arg399Gln GGvs AA	Slope	0.0161113	0.0407762	0.40	0.696	−0.0672855—0.099508
	Bias	−1.085402	0.8032467	−1.35	0.187	−2.728226—0.5574217
Arg194Trp CCvs TT	Slope	0.0710443	0.055634	1.28	0.226	−0.0501718—0.1922603
	Bias	−2.741172	1.294758	−2.12	0.056	−5.562207—0.079863
Arg280His GGvs GA	Slope	0.0252937	0.009486	2.67	0.056	−0.0010435—0.051631
	Bias	−3.029606	0.7672345	−3.95	0.017	−5.159791—0.8994219

CI = confidence interval, XRCC1 = X-ray repair cross complementation 1.

### 3.5 Ethics and dissemination

The literature collected by the Institute is derived from published academic literature in a professional network database, and the data used in statistical analysis can be obtained from these publicly published papers, so the study does not require ethical approval.

## 4 Discussion

The occurrence and development of cancer is the result of a combination of multiple factors, including genetic inheritance, hormone levels, inflammatory factors and dietary habits. Over the past decade, advances have been made in the pathogenesis of gynecologic tumors and in anticancer therapies. However, the 5-year survival rate is still very low, so it is important to find new molecular markers that can be used to predict the risk of cancer. XRCC1 gene encodes a homonymous protein that is an important functional protein in the process of single-strand DNA damage. Mutation of the XRCC1 allele is associated with reduced DNA repair ability and prolonged cell cycle. XRCC1Arg399Gln non-synonymous mutations cause amino acid sequence changes that affect protein function and DNA repair ability, and may affect the interaction with other DNA repair proteins, leading to an increased risk of tumor.^[55]^ Wu^[56]^ detected XRCC1 mRNA expression in peripheral blood of patients with ovarian cancer in the platinum sensitive group (19 cases), part of the platinum sensitive group (25 cases) and the platinum resistant group (22 cases). Results: The expression level of XRCC1 protein in platinum-resistant group was higher than that in platinum-sensitive group (*P* < .05). This indicated that XRCC1 gene expression in peripheral blood may affect the sensitivity of cisplatin chemotherapy for ovarian cancer.

The current polymorphism studies of XRCC1 mainly focus on 3 non-synonymous mutations SNPs, which are Arg194Trp, Arg399Gln and Arg280His respectively. The relationship between XRCC1 polymorphism and susceptibility to gynecological malignant tumors has been reported for many times, but negative reports are also common.^[57–60]^ Therefore, there is still no consensus on the relationship between XRCC1 polymorphism and gynecological malignant tumors. Different research designs, different test methods, the number of samples and the difference in population distribution will inevitably affect the experimental conclusions. As a powerful tool, meta-analysis can overcome the above factors, analyze data and conclusions, and provide a basis for subsequent research.

Heterogeneity is an important part of meta-analysis, and it is very important to understand the source of heterogeneity,^[61]^ which can often provide us with ideas for solving problems. In our meta-analysis, heterogeneity was mainly derived from Khokhrin, Sonali Verma, Jakubowska.^[31,35,50]^ All 3 studies were of ovarian cancer in European, non-Asian populations, with population as a major factor.

In the TCGA dataset, it can be found that XRCC1 has the highest mutation rate in Uterine Corpus Endometrial Carcinoma samples, Cervical squamous cell carcinoma and endocervical adenocarcinoma has a relatively high mutation rate, and ovarian cancer has a low mutation rate, indicating that the mutation of this gene may lead to endometrial cancer and cervical cancer. The deficiency of this study is that there are only 6 references on endometrial cancer included, of which only 4 are in line with Hardy-weinberg equilibrium, and the sample population is all Asian. To solve the above problems, more samples and multi-ethnic studies are needed in the follow-up studies.

XRCC1 Arg399Gln, Arg194Trp, Arg280His single nucleotide polymorphisms were associated with gynecologic cancer susceptibility. Arg399Gln was statistically significant with cervical cancer susceptibility. Arg194Trp was statistically significant for susceptibility to endometrial cancer. XRCC1 genotype detection at each site is expected to be a molecular marker for gynecologic cancer screening.

## Acknowledgments

This research was completed under the guidance of my tutor Professor Li Li, which provided me with great help and support in terms of research topic selection, research ideas, statistical methods, etc. I would like to thank my tutor for his guidance and tolerance and for his suggestions on the revision of my article.

## Author contributions

**Data curation:** Xue Qin Zhang.

**Investigation:** Xue Qin Zhang.

**Resources:** Xue Qin Zhang.

**Supervision:** Li Li.

**Writing – original draft:** Xue Qin Zhang.

**Writing – review & editing:** Li Li.
